# Identification and Analysis of the Tegument Protein and Excretory-Secretory Products of the Carcinogenic Liver Fluke *Clonorchis sinensis*

**DOI:** 10.3389/fmicb.2020.555730

**Published:** 2020-09-23

**Authors:** Yunliang Shi, Kai Yu, Anli Liang, Yan Huang, Fangqi Ou, Haiyan Wei, Xiaoling Wan, Yichao Yang, Weiyu Zhang, Zhihua Jiang

**Affiliations:** ^1^Institute of Parasitic Disease Control and Prevention, Guangxi Zhuang Autonomous Region Center for Disease Control and Prevention, Nanning, China; ^2^Guangxi Key Laboratory for the Prevention and Control of Viral Hepatitis, Guangxi Zhuang Autonomous Region Center for Disease Control and Prevention, Nanning, China; ^3^College of Animal Science and Technology, Guangxi University, Nanning, China; ^4^Xiangsihu College of Guangxi University for Nationalities, Nanning, China

**Keywords:** *C. sinensis*, proteomics, tegument, biotinylation, cholangiocarcinoma

## Abstract

Liver fluke proteins, including excretory-secretory products (ESPs) and tegument proteins, are critical for the pathogenesis, nutrient metabolism, etiology and immune response of liver cancer. To understand the functions of various proteins in *Clonorchis sinensis* physiology and human clonorchiasis, the ESPs and tegument proteins of *C. sinensis* were identified. Supernatants containing ESPs from adult *C. sinensis* after culture for 6 h were harvested and concentrated. The tegument was detached using a freeze/thaw method and successively extracted using various extraction buffers. The outer surface proteins of *C. sinensis* were labeled with biotin, and the biotinylated proteins were purified. The ESP, tegument and labeled outer surface proteins were identified and analyzed by high-resolution LC-MS/MS. The identified proteins were compared with those of other flukes, and the protein functions associated with pathogenesis, carcinogenesis and potential vaccine antigens and drug targets were predicted and analyzed. A total of 175 proteins were identified after the 6-h culture of *C. sinensis* ESPs. A total of 352 tegument proteins were identified through sequential solubilization of the isolated teguments, and a subset of these proteins were localized to the surface membrane of the tegument by labeling with biotin. Thirty identified proteins, including annexins, actin and tetraspanins, were identified as potential immunomodulators and promising vaccine antigens. Interestingly, among the 352 tegument proteins, as many as 155 were enzymes, and most were oxidoreductases, hydrolases or transferases. A comparison of the outer surface proteins of *C. sinensis* with those of other flukes indicated that flukes have some common outer surface proteins, such as actin, tetraspanin, glyceraldehyde-3-phosphate dehydrogenase (GAPDH) and annexin. Granulin, thioredoxin peroxiredoxin, carbonyl reductase 1 and cystatin were identified in the *C. sinensis* proteome and predicted to be related to liver disease and cancer. The analysis of the *C. sinensis* proteome could contribute to a more in-depth understanding of complex parasite-host relationships, improve the diagnosis of clonorchiasis and benefit research on the pathogenesis and development of novel interventions, drugs and vaccines to control *C. sinensis* infection.

## Introduction

*Clonorchis sinensis* is a food-borne zoonotic parasite, and humans infected with the liver fluke *C. sinensis* constitute a serious public health problem in many parts of southeast Asia, including China, Korea, and Vietnam (Lun et al., 2005). Approximately 35 million people are infected worldwide. People infected with *C. sinensis* mainly digest raw fish contaminated with metacercaria. *C. sinensis* adults, which dwell in bile ducts, cause subclinical or clinical hepatic and biliary disease with biliary epithelial hyperplasia, periductal fibrosis, and cystic changes in the ducts and even facilitate the development of cholangiocarcinoma (CCA) (Lee et al., 1993; Olnes and Erlich, 2004; Fried et al., 2011). Both clinical observations and epidemiological evidence strongly implicate *C. sinensis* infection in the pathogenesis of CCA (Choi et al., 2006, 2010; Shi et al., 2017).

Excretory-secretory products (ESPs) are antigens that include a complex mixture of secreted proteins and some extracellular vesicles (EVs) secreted through the parasite’s tegument and products from oral openings or the gut of the parasite. ESPs are involved in parasite–host interactions and play key roles in the migration of *C. sinensis* adults to intrahepatic bile ducts and in the development of liver damage and even CCA (Kim Y. J. et al., 2009; Pak et al., 2009, 2019). Knowledge of the components of ESPs has value in rendering a diagnosis (Kang et al., 2019; Kim et al., 2019). Identification of the composition of the ESPs could provide attractive materials for the identification of causative agent candidates and new drug targets. However, the proteomic profiles of the ESPs in *C. sinensis* remain obscure.

The tegument constitutes the outermost surface of the parasite and is composed of a syncytial layer. The outer surface proteins of the tegument are enriched in enzymes and glycans and thus enable the parasite to directly contact immune cells, antibodies and various cytokines. Tegument proteins are thought to play key roles in parasite reproduction, signal transduction, nutrition intake, pathogenesis and the modulation of host immune responses and are therefore promising new drug targets and vaccine candidates (Dalton et al., 2004; Jones et al., 2004; Loukas et al., 2007). Previous studies have identified tegument and outer surface proteins (labeled with biotin) of *Opisthorchis viverrini* (Mulvenna et al., 2010b), *Schistosoma mansoni* (van Balkom et al., 2005; Braschi and Wilson, 2006; Braschi et al., 2006; Sotillo et al., 2015), *Schistosoma japonicum* (Mulvenna et al., 2010a), *Streptococcus bovis* (de la Torre-Escudero et al., 2013), and *Fasciola hepatica* (Wilson et al., 2011; Ravidà et al., 2016), and some important proteins have been revealed to be useful for immunologic diagnosis, drug targeting and vaccine development (Mossallam et al., 2015; Chen et al., 2016). Here, we characterize both the ESPs and the tegument proteins of *C. sinensis* through tryptic digestion coupled with high-resolution LC-MS/MS and describe the proteins that have potential roles in pathogenesis and carcinogenesis and that can serve as potential vaccine antigens and drug targets.

## Materials and Methods

### Preparation of *C. sinensis* ESP Proteins

*Clonorchis sinensis* ESPs were obtained using established methods (Kim Y. J. et al., 2009). In brief, *C. sinensis* metacercariae were obtained from naturally infected cyprinoid fish in Hengxian County, Guangxi Province, China. The fish were digested with pepsin-HCl and washed, and the metacercariae were collected and used to infect rats (*Mesocricetus auratus*) via stomach incubation. The rats were maintained at the animal research facility of Guangxi Disease Prevention and Control Center using protocols approved by the Guangxi Disease Prevention and Control Center Animal Ethics Committee. Fresh adult flukes were recovered from the bile ducts of euthanized rats infected for 40 days and washed several times with normal saline containing penicillin (200 U/ml) and streptomycin (200 U/ml). Dead or dying flukes were discarded, and the ESPs were prepared by incubating viable flukes in modified PBS (Invitrogen) containing penicillin (100 U/ml) and streptomycin (100 U/ml) at 37°C in 5% CO_2_. Supernatants containing the ESPs were collected after 6 h, centrifuged at 12,000 × *g* and 4°C for 30 min to remove the parasites’ eggs from the media, concentrated to 100–250 μg/ml using a 2-kDa spin concentrator (Sartorius, Germany) and aliquoted for storage at −80°C.

### Preparation of Whole Tegument Proteins

The whole tegument (WT) proteins of *C. sinensis* were prepared as previously described for *O. viverrini* (Mulvenna et al., 2010b). Briefly, fresh adult flukes were obtained from the infected rats as described above. Healthy and undamaged flukes were used for tegument detachment using a freeze/thaw method, successively extracted with various extraction buffers and pooled together (Mulvenna et al., 2010b). The extractions were precipitated with three volumes of acetone at −20°C.

### Preparation of Biotinylated Tegument Proteins

The biotinylated tegument proteins (BTs) of *C. sinensis* were prepared as previously described for *O. viverrini* (Mulvenna et al., 2010b). Briefly, undamaged flukes were labeled with 1 mM sulfosuccinimidyl-6-[biotinamido]hexanoate (EZ-Link^TM^ sulfo-NHS-LC-biotin, Pierce) in Hank’s balanced salt solution (HBSS), and teguments were removed and solubilized. The extractions were combined and incubated with 240 μl of streptavidin-agarose beads (GE Healthcare, United Kingdom). The proteins bound to the streptavidin were then eluted with 2% SDS, the supernatants were combined, and the proteins were precipitated using three volumes of acetone at −20°C.

### Fluorescence Microscopy-Based Observation of Surface Biotinylation

To observe the surface biotinylation of *C. sinensis*, the flukes were biotinylated with 1 mM EZ-Link sulfo-NHS-SS-biotin, as described previously, washed three times in HBSS, incubated with streptavidin-FITC for 30 min at room temperature and washed three times with HBSS. Unlabeled (no biotin) flukes incubated with streptavidin-FITC alone and biotin-labeled flukes not incubated with streptavidin-FITC were used to measure the autofluorescence and as negative controls. The samples were visualized using a Zeiss Axio Imager M2 ApoTome fluorescence microscope (Zeiss, Germany) equipped with an AxioCam MRN at 40 × magnification.

### Protein Purification Prior to Digestion

The crude protein precipitate of *C. sinensis* was added to 1:50 (W/V) lysis buffer (2 mM EDTA, 8 M urea, 10 mM DTT and 1% protease inhibitor cocktail), sonicated for 1–2 min, and centrifuged at 13,000 × *g* and 4°C for 10 min to remove debris. The supernatant was collected and precipitated using three volumes of acetone for 3 h at −20°C. After centrifugation at 4°C and 12,000 × *g* for 10 min, the protein deposit was redissolved in urea buffer [8 M urea and 100 mM triethylammonium bicarbonate (TEA)]. The concentration of the protein was determined using a modified Bradford protein assay kit (Sangon Biotech, China) according to the manufacturer’s instructions.

### Trypsin Digestion and Peptide Desalting

For trypsin digestion, 10 μg of the sample proteins was reduced with 10 mM DTT at 37°C for 60 min and alkylated with 25 mM iodoacetamide (IAM) at room temperature for 30 min in darkness. The urea concentration of the protein sample was diluted to less than 2 M by adding 100 mM TEAB. The protein pool of each sample was digested overnight at 37°C with sequencing-grade modified trypsin at a protein:trypsin mass ratio of 50:1 and then subjected to further digestion for 4 h with trypsin at a ratio of 100:1. After trypsin digestion, the peptides were desalted on a Strata X SPE column and vacuum-dried.

### High-Resolution LC-MS/MS Analysis

The experiment was performed with a NanoLC 1000 LC-MS/MS system using a Proxeon EASY-nLC 1000 coupled to a Thermo Fisher LTQ-Orbitrap Elite mass spectrometer. There is only one biological replicate of each fraction WT, BT, and ESP, and proteomic run of each fraction was performed only one. The trypsin-digested fractions were reconstituted in 0.1% FA, and 2 μg of each sample was directly loaded onto a reversed-phase precolumn (Acclaim PepMap^®^100 C18, 3 μm, 100 Å, 75 μm × 2 cm) with 100% solvent A (0.1 M acetic acid in water) at 5 μl/min. The peptides eluted from the trap column were loaded onto a reversed-phase analytical column (Acclaim PepMap^®^ RSLC C18, 2 μm, 100 Å, 50 μm × 15 cm). The gradient program was as follows: 15 to 35% solvent B (0.1% FA in 98% ACN) over 45 min, 35 to 98% solvent B over 5 min and 98% solvent B for 5 min; a constant flow rate of 250 nl/min was maintained throughout the program. The eluent was sprayed with an NSI source with a 1.8-kV electrospray voltage and then analyzed by tandem mass spectrometry (MS/MS) using an LTQ-Orbitrap Elite instrument. The mass spectrometer was operated in the data-dependent mode, which involves automatically switching between MS and MS/MS. Full-scan MS spectra (from m/z 350 to 1800) were acquired with the Orbitrap at a resolution of 60,000. Ion fragments were detected at a resolution of 15,000, and the 20 most intense precursors were selected for HCD fragmentation at the collision energy of 38% in the MS survey scan with 45.0-s dynamic exclusion.

### Database Searching and Data Processing

Using the Sequest software integration tool in Proteome Discoverer (version 1.3, Thermo Scientific), the MS/MS raw data were searched against a *C. sinensis* database downloaded from the UniProt database with additional contaminant and host proteins. Trypsin was chosen as the enzyme, and two missed cleavages were allowed. Carbamidomethylation (C) was set as a fixed modification, and oxidation (M) and acetylation in the N-Term were set as variable modifications. The searches were performed using a peptide mass tolerance of 10 ppm, a product ion tolerance of 0.02 Da, and a false discovery rate (FDR) of 5% against a decoy database, and single peptide matches were included in the lists of accepted protein identifications. Comparisons were made with the results obtained using two additional database searching software programs (Mascot and MaxQuant) with the same parameter settings.

### Bioinformatics Analysis

The subcellular localization of the identified proteins was predicted using WoLF PSORT software, and the sequences were predicted using an updated version of PSORT/PSORT II in WoLF PSORT (Paul et al., 2007) and literature searches. The enriched proteins identified in a Gene Ontology analysis were classified by GO annotation. The identified proteins (UniProt ID) were annotated with the UniProt-GOA database^1^ and then classified via Gene Ontology annotations based on three categories: biological processes, cellular components and molecular functions. For each category, a two-tailed Fisher’s exact test was employed to test the enrichment of the differentially expressed proteins compared with all the identified proteins. Correction for multiple hypothesis testing was performed using standard FDR correction methods such as the Benjamini–Hochberg procedure. Differences in the GO-enriched proteins with a corrected *p*-value < 0.05 were considered significant.

A pathway enrichment analysis was also conducted. The Encyclopedia of Genes and Genomes (KEGG)^2^ was used to identify the enriched pathways based on a two-tailed Fisher’s exact test to determine the enrichment of the identified proteins in each fraction compared with all the identified proteins. First, the KEGG online service tool KAAS^3^ was used to annotate the proteins according to the description in the KEGG database. The annotation results were mapped to the KEGG pathway database using the KEGG online KEGG mapper service tool. Correction for multiple hypothesis testing was performed using standard FDR control methods and the Benjamini–Hochberg multiple correction procedure. Differences in pathway proteins with a corrected *p*-value < 0.05 were considered significant. The proteins were classified into hierarchical pathway categories according to terms in the KEGG website.

## Results

### Proteins Identified Among the *C. sinensis* ESPs

**FIGURE 1:**
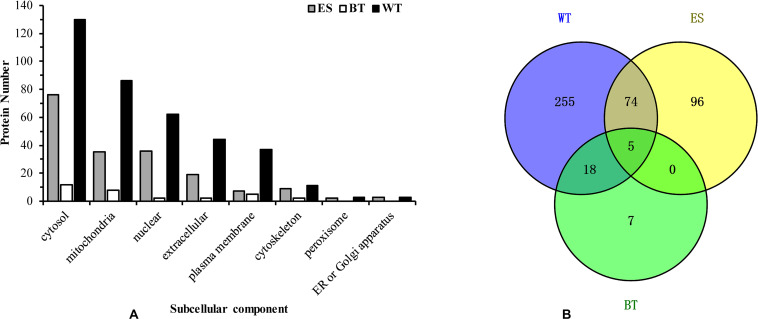
Subcellular location of the *Clonorchis sinensis* proteins. Bar graph showing the abundance of excretory-secretory products, tegument proteins, and outer surface proteins at different subcellular locations **(A)**. Venn diagrams of common excretory-secretory product proteins, tegument proteins and outer surface proteins **(B)**.

Approximately 120 μg of *C. sinensis* ESPs was obtained by culturing worms for a period of 6 h in PBS. The proteomic characterization of this mixture yielded 175 unique proteins to be identified (Supplementary Table 1). Using peptide-spectrum matches (PSMs) as a guide for determining the relative abundance of the identified proteins (James et al., 2015), the top five most abundant proteins were found to be basement membrane-specific heparan sulfate proteoglycan core protein, glutathione transferase, dynein, retinal dehydrogenase 1 and myoglobin. Some proteases, such as glutathione transferase, dehydrogenase and cysteine protease, were also among the top 10 most common proteins in the samples. The prediction of the subcellular localization of the proteins showed that most of the proteins were located in the cytosol (68 proteins), nucleus (23 proteins), mitochondria (22 proteins), and extracellular space (19 proteins) (Figure 1A). Seventeen proteins were uncharacterized proteins with no known homology.

### Identification of *C. sinensis* Tegument Proteins

The tegument of approximately 200 adult *C. sinensis* was stripped and sequentially extracted, and a proteomic analysis of these fractions yielded 352 unique protein read identifies (Supplementary Table 2). The values of the PSMs indicated that the top ten most abundant proteins were nesprin-1, dynein, myosin, basement membrane-specific heparan sulfate proteoglycan core protein, and voltage-dependent anion channel protein 2. The majority of the tegument proteins were predicted to be localized in the cytosol (36.9%), mitochondria (17.9%), extracellular space (11.9%), nucleus (11.3%), and plasma membrane (10.5%). Forty-two extracellular and 37 plasma membranes were identified (Figure 1A), and 17 proteins were uncharacterized proteins with no homology to other proteins in the GenBank database.

### Identification of BTs After Labeling the Surface of Live Worms

**FIGURE 2:**
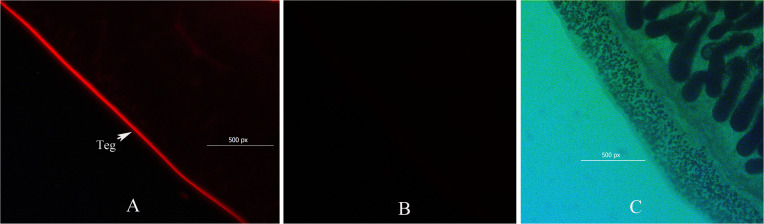
Immunofluorescence micrograph showing the surface biotinylation of adult *Clonorchis sinensis*. Transformed worms were incubated with biotin and probed with streptavidin-FITC. The short-chain thiol-cleavable biotin was incorporated only into the outer surface of the tegument **(A)**. Streptavidin-FITC did not bind to the tegument of non-biotinylated worms **(B)**. Bright field showing worm biotin probed with streptavidin-FITC **(C)**.

The localization of biotin on the labeled worms, which was observed by fluorescence microscopy, showed a clear zone of labeling around the exterior of the worm, and the biotin did not penetrate into the tegument membrane or the gut membrane (Figure 2). Approximately 200 live and undamaged adult *C. sinensis* worms presented biotin labeling on their outer surface. Thirty proteins were identified among the biotin-labeled proteins, and according to the PSM values, the most abundant proteins were actin, 70-kDa heat shock protein, prostaglandin-H2, glutamate dehydrogenase, thioredoxin peroxidase, succinate dehydrogenase and the Ras-related protein Rab-27B. Fourteen cytoskeletal proteins were identified in the biotinylated tegument fraction, and these included glyceraldehyde-3-phosphate dehydrogenase (GAPDH), elongation factor 1-alpha, and prostaglandin-H2. Seven mitochondrial proteins, including glutamate dehydrogenase (NAD(P)+), voltage-dependent anion channel protein 2, succinate dehydrogenase and uridine phosphorylase, and five plasma membrane-associated proteins, including tetraspanin, solute carrier family 25, glycerol kinase, ADP/ATP carrier and sodium/glucose cotransporter 4, were also found in this fraction (Table 1).

**TABLE 1:** Proteins identified from the *Clonorchis sinensis* biotinylated tegument preparation fraction.

GI	SC	CO	UP	Description	Ov	Sm	Sj	Sb	Fh
H2KRT8	27.89	40.69	1	Actin beta/gamma 1	+	+	+	+	+
G7YCA9	16.99	35.48	1	Actin beta/gamma 1					
H2KVT7	9.20	34.27	3	Prostaglandin-H2 D-isomerase	−	−	−	−	+
H2KPM6	0.00	9.65	1	Annexin A7	+	+	+	+	+
H2KPI5	1.84	5.28	1	Voltage-dependent anion channel protein	+	+	+	−	+
H2KPE2	3.97	21.07	1	Glyceraldehyde-3-phosphate dehydrogenase	+	+	+	+	−
H2KNU1	7.01	18.97	2	Tegument antigen	−	−	−	−	−
G7YTI6	0.00	15.60	1	Ras-related protein Rab-27B	−	−	−	−	−
G7YIE7	0.00	23.39	1	Heat shock 70-kDa protein	−	+	+	+	+
G7YD93	4.27	12.98	2	Fructose-bisphosphate aldolase	−	+	−	+	−
G7YBA9	0.00	12.77	1	Beta-2-syntrophin	−	−	−	−	−
G7YA24	1.90	8.97	1	Uridine phosphorylase	−	−	−	−	−
E2JF28	0.00	13.85	1	Thioredoxin peroxidase	+	−	+	+	−
1 WA65	6.19	14.10	2	Elongation factor 1-alpha	−	−	+	−	−
G7YK67	2.16	6.77	1	Fatty-acid amide hydrolase	−	−	−	−	−
G7YGH4	3.00	17.31	1	Uncharacterized protein	−	−	−	−	−
G7Y7T6	5.53	19.74	1	Glutathione S-transferase	−	−	+	+	−
G7YW97	73.79	32.46	1	Propionyl-CoA carboxylase alpha chain	−	−	−	−	+
G7YT72	4.74	12.92	1	Endophilin-B1	−	+	+	−	+
G7YS59	2.49	15.73	1	Pyruvate carboxylase	+	−	−	−	+
G7YQG7	0.00	14.29	2	Succinate dehydrogenase	+	−	−	−	+
G7YLC0	6.86	13.61	1	Glutamate dehydrogenase	+	−	−	+	+
G7YHC3	0.00	15.35	1	Uncharacterized protein	−	−	−	−	−
G7YFK3	10.36	12.52	2	Propionyl-CoA carboxylase beta chain	−	−	−	−	+
G7YFT8	2.06	21.43	1	Fatty acid-binding protein type 3	−	+	−	+	−
A7XWR4	14.68	21.19	2	ADP/ATP carrier	+	+	+	+	−
G7YXC3	3.26	6.52	1	Solute carrier family 25 member	−	−	−	−	−
G7YB65	2.21	8.21	1	Tetraspanin	+	+	+	+	+
H2KSG0	2.11	8.32	1	Glycerol kinase	+	−	−	−	−
H2KVD0	1.99	3.03	1	Sodium/glucose cotransporter 4	−	−	−	−	−

*The abbreviations are defined as follows: GI, gene accession; UP, unique peptide; SC, spectral count; CO, percent coverage. The presence and presence of the indicated protein in the outer surface of *O. viverrini* (Mulvenna et al., 2010b), *S. mansoni* (van Balkom et al., 2005; Braschi and Wilson, 2006; Braschi et al., 2006; Sotillo et al., 2015), *S. japonicum* (Mulvenna et al., 2010a), *S. bovis* (de la Torre-Escudero et al., 2013), and *F. hepatica* (Wilson et al., 2011; Ravidà et al., 2016) is denoted with a “+” and “−,” respectively.*

The outer surface proteins of *C. sinensis* were compared with the identified outer surface proteins of *O. viverrini* (Mulvenna et al., 2010b), *S. mansoni* (van Balkom et al., 2005; Braschi and Wilson, 2006; Braschi et al., 2006; Sotillo et al., 2015), *S. japonicum* (Mulvenna et al., 2010a), *S. bovis* (de la Torre-Escudero et al., 2013), and *F. hepatica* (Wilson et al., 2011; Ravidà et al., 2016; Table 1), and actin, tetraspanin, GAPDH and annexin, among other proteins, were found in all of these flukes. The comparison of *C. sinensis* and *O. viverrini*, which have similar lifecycles, revealed that these worms have a number of the same outer surface proteins, such as actin, ADP/ATP carrier, NAD(P)+, GAPDH, pyruvate carboxylase, tetraspanin, glycerol kinase, thioredoxin peroxidase, succinate dehydrogenase and annexin (Table 1). However, some proteins, such as sodium/glucose cotransporter 4 and fatty-acid amide hydrolase 1, appeared to be specific to *C. sinensis* and were not found in *O. viverrini*, *S. mansoni*, *S. japonicum*, *S. bovis*, and *F. hepatica* (Table 1).

### Comparison of the Identified ESPs, WT Proteins and BTs

The mass spectrometry data were submitted to ProteomeXchange and were given the accession number PXD019116. The subcellular localization of the identified proteins was predicted using WoLF PSORT software, and the sequences were predicted with an updated version of PSORT/PSORT II in WoLF PSORT. A more in-depth analysis based on the subcellular localization showed some differences in the expression profiles of the proteins in the ESP, WT, and BT fractions. Most of the ESP proteins are cytoplasmic, nuclear or extracellular proteins, whereas the WT and BT fractions mostly contain cytoplasmic, nuclear, mitochondrial and plasma membrane proteins (Figure 1A). A Venn diagram of the identified proteins in the ESP, WT and BT fractions was constructed. Among the identified proteins, 30, 352, and 175 were only found in the BT, WT, and ESP fractions, respectively, five proteins were identified in all three fractions, 23 proteins were found in both the BT and WT fractions, five proteins were found in both the BT and ESP fractions, and 79 proteins were found in both the WT and ESP fractions (Figure 1B).

### Analyses of the Biological Processes and Pathways of the Identified Proteins

**FIGURE 3:**
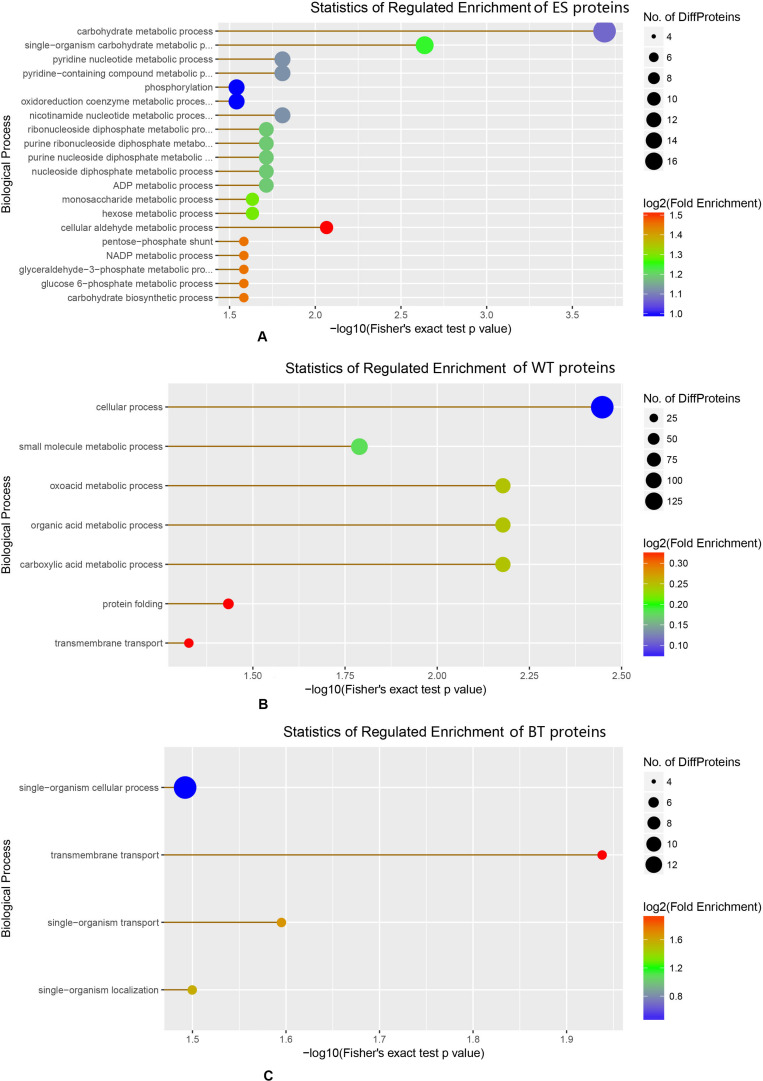
Enrichment of different biological proteins among the excretory-secretory product, tegument protein, and outer surface protein fractions. **(A)** Statistics of regulated enrichment of excretory-secretory protein. **(B)** Statistics of regulated enrichment of tegument protein. **(C)** Statistics of regulated enrichment of biotinylated tegument protein.

**FIGURE 4:**
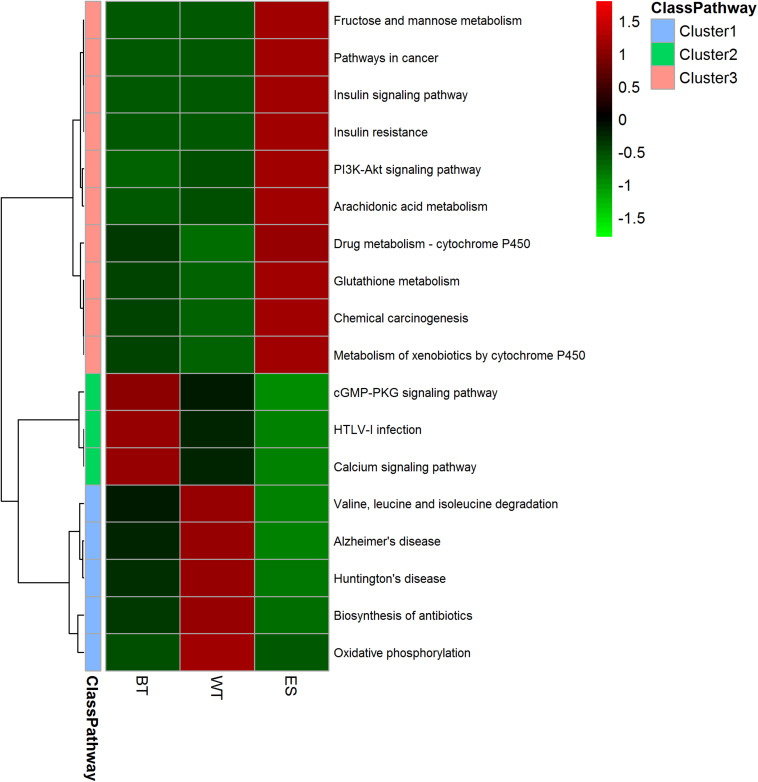
Heatmap showing the pathway clusters of excretory-secretory products, tegument proteins and outer surface proteins. The abundance of the proteins is represented by different colors and compared among the three protein fractions. The proteins were grouped into 10 different Gene Ontology (GO) categories based on GO annotations and six cellular location categories predicted with UniLoc and literature searches. The clustering was performed using Euclidean distances.

The GO annotation results showed that the ESPs, WT proteins and outer surface proteins were associated with different biological processes. The ESPs were found to be associated with complicated biological processes, whereas the WT and outer surface proteins were associated with substantially fewer biological processes, mainly cellular, metabolic and transmembrane transport processes (Figure 3). The identified proteins in the ESP fraction were predicted to be mainly associated with the following pathways: chemical carcinogenesis, metabolism of xenobiotics by cytochrome 450, drug metabolism-cytochrome P450, insulin signaling, glutathione metabolism and fructose and mannose metabolism. The analysis also revealed that the identified WT proteins were mainly associated with oxidative phosphorylation, valine/leucine and isoleucine degradation, biosynthesis of antibiotics, Huntington’s disease and Alzheimer’s disease, whereas the BTs were mainly associated with calcium, cGMP-PKG, Hippo signaling and the HTLV-I infection pathway (Figure 4).

## Discussion

The genome and transcriptome of *C. sinensis* have been revealed (Young et al., 2010; Wang et al., 2011b; Huang et al., 2013), and this knowledge greatly contributes to our understanding of different processes, such as parasite development, nutrition, immune evasion, and modulation. The completed *C. sinensis* genome was sequenced and annotated, and approximately 16,000 protein-coding gene models have been predicted (Wang et al., 2011b); in addition, a large number of secreted and tegument proteins have been identified and are available in GenBank, which makes the search for *C. sinensis* proteins accessible and reliable. Several reports have indicated that the ESPs of *C. sinensis* might play key pathogenic roles in the development of hepatic and bile duct diseases (Kim H. G. et al., 2009, Kim et al., 2019). However, few studies have examined the identities of *C. sinensis* ESPs. Zheng et al. (2011, 2013) identified 110 and 267 proteins in *C. sinensis* ESPs by LC–MS/MS after culture for 0–48 h (Zheng et al., 2011) and 0–6 h (Zheng et al., 2013), respectively. In the current study, we identified 175 ESPs, and the differences in the number of identified proteins might be associated with the protocol used for protein preparation, the protein search software program and the parameter settings. In our research, we used liquid enzyme hydrolysis, not gel electrophoresis, to effectively reduce the loss of protein. In the protein search, we set a rigorous quality error for the secondary spectrum. Because not all details of the proteins identified by Zheng et al. (2011) and Zheng et al. (2013) are included in the papers and published databases, we are unable to perform an in-depth comparison. However, some of the proteins, such as enolase, actin, heat shock protein, and Ras-related protein, were found in both the previous investigations and the current study.

The ESPs of parasites include molecules that might be useful in the development of diagnostics, vaccines, and drug therapies. In the current study, a total of 175 proteins were identified after 6 h of the *in vitro* culture of *C. sinensis*, and these included some important proteins, such as enolase, which is a cytosolic glycolytic enzyme that localizes to the cell surface and tegument in helminths. In some schistosome species (*S. bovis* and *S. japonicum*), enolase has been identified at the host-parasite surface and serves as a PLMG activator (de la Torre-Escudero et al., 2010; Yang et al., 2010; Figueiredo et al., 2015). In *C. sinensis*, enolase is an important glycolytic enzyme required for parasite growth (Wang et al., 2011a), and knockdown of the enolase gene in *C. sinensis* increases worm mortality (Wang et al., 2014a). Cathepsins have been identified as good diagnostic antigens, in addition, it has been associated with nutrient uptake (Na et al., 2008; Ma et al., 2019), and several cysteine protease subtypes, such as cathepsin A, B, D, E, and F, have been found in *C. sinensis* (Wang et al., 2011b; Kang et al., 2019); After 6 h of *C. sinensis* culture, we identified cathepsin C in the ESP fraction, and cathepsin C was specifically distributed in the suckers (oral and ventral suckers), eggs, vitellarium, intestines, and testis of *C. sinensis*, and it plays roles in the digestion of host proteins, nutrition assimilation, and immune invasion. Furthermore, cathepsin C might be a potential diagnostic antigen and drug target against *C. sinensis* infection (Liang et al., 2014).

In the ESPs of flukes that should contain some EVs, EVs can be purified from the ESPs by differential ultracentrifugation, and the EVs of some trematodes have been identified from the secretory products (Marcilla et al., 2012; Chaiyadet et al., 2015; Davis et al., 2019). Previous studies have shown that EVs of trematodes contain some of the proteins previously identified as components of ESPs; specifically, more than half of the molecules identified in the secretomes of *Echinostoma caproni* and *F. hepatica* are found in these exosome-like vesicles (Marcilla et al., 2012). At present, the EVs of *C. sinensis* have not been studies, and we could not identify overlapping proteins between ESPs and EVs.

Previous studies have identified the tegument proteins of *O. viverrini* (Mulvenna et al., 2010b), *F. hepatica* (Wilson et al., 2011), *S. japonicum* (Mulvenna et al., 2010a), *S. mansoni* (van Balkom et al., 2005; Braschi and Wilson, 2006; Braschi et al., 2006; Sotillo et al., 2015), and *S. bovis* (de la Torre-Escudero et al., 2013). Using the techniques described, the WT of *C. sinensis* was removed by freeze-thawing and successively extracted with buffers of increasing solubilization strength, which enabled the enrichment of membrane-associated proteins, and proteins within and exposed at the tegument surface were identified. Of the 352 identified *C. sinensis* tegument proteins identified, many were proteases, and these proteases might be closely related to nutrient uptake, immune escape and migration. A cathepsin B-like cysteine proteinase was identified in *C. sinensis*, and a novel tegument cathepsin B was identified in *S. mansoni* (Caffrey et al., 2002). Cathepsin B-like cysteine proteinase can decompose the skin tissue and help the cercaria penetrate into skin (Wilson and Coulson, 2006). The cathepsin B-like cysteine proteinase in *C. sinensis* might facilitate larval migration. The identified enolases have been previously studied and are expressed during the adult worm, metacercaria, cercaria and egg life stages of *C. sinensis*. Enolases exhibit a significantly higher expression level at the adult stage and are deposited on the tegument of the adult worm and cyst wall of the metacercaria. Enolases might play key roles in the growth of parasites and might be promising oral vaccine candidates (Wang et al., 2011a, 2014b). Most of the identified proteases were oxidoreductases, hydrolases and transferases, which indicated that the tegument is key for nutrient uptake and energetic metabolism.

Whole fluke biotinylation is a technique that has been used to label and identify the outer surface of tegument proteins. The reagent used in this study (Sulfo-NHS-LC-biotin) can react with exposed primary amines and preferentially labels surface-exposed proteins. Previous studies used biotin labeling to identify the outer surface proteins of *O. viverrini* and *S. mansoni* (Mulvenna et al., 2010b; Sotillo et al., 2015). The outer surfaces of *O. viverrini* (Mulvenna et al., 2010b), *S. mansoni* (van Balkom et al., 2005; Braschi and Wilson, 2006; Braschi et al., 2006; Sotillo et al., 2015), *S. japonicum* (Mulvenna et al., 2010a), *S. bovis* (de la Torre-Escudero et al., 2013), and *F. hepatica* (Wilson et al., 2011; Ravidà et al., 2016) have been identified, and the number of identified outer surface proteins ranges from 22 to 45. Thirty proteins were identified from the biotin-labeled outer surface of *C. sinensis*, and various proteins, such as actin, tetraspanin, GAPDH and annexin, were found in *C. sinensis*, *O. viverrini*, *S. mansoni*, *S. japonicum*, *S. bovis*, and *F. hepatica* (Table 1), which indicates that these outer surface proteins of flukes might have similar functions. Two tetraspanins (TSP1 and TSP2) were identified in the outer surface of *S. mansoni* and *S. japonicum* and function in tegument renewal (Tran et al., 2010), and these have been found to be promising vaccine candidates to protect against schistosomiasis (Tran et al., 2010; Chen et al., 2016). Tetraspanin 2 was previously found in *C. sinensis* (Kim et al., 2011), but the function of tetraspanins in *C. sinensis* remains unknown. Annexin is another important protein identified in the *C. sinensis* tegument. In *S. mansoni*, annexin binds to the surface membranes of the tegument in a calcium-dependent manner and is considered a potential vaccine candidate (Tararam et al., 2010). Annexin B30, which is composed of four annexin repeats, was previously found in *C. sinensis* and is distributed in the tegument, intestine, and eggs of adult worms and in the tegument and vitellarium of metacercaria. This protein plays a role in adjusting the host immune response during *C. sinensis* infection (He et al., 2014).

Considering the different biological fluids in which each parasite resides, such as the blood for schistosomes and the bile ducts for *O. viverrini* and *C. sinensis*, the tegument proteins of these two different flukes are expected to show some differences, particularly in the composition of the proteins exposed to hosts, which might be relevant to the host immune response and to nutrient uptake and metabolism. Pyruvate carboxylase, succinate dehydrogenase and glycerol kinase were found only on the outer surface of *C. sinensis* and *O. viverrini* but not in *Schistosoma*. These three enzymes are associated with nutrition absorption and energy metabolism. Pyruvate carboxylase is an anaplerotic enzyme that plays important roles in various cellular metabolic pathways, including fatty acid synthesis, amino acid synthesis, gluconeogenesis and glucose-induced insulin secretion. This enzyme is involved in tumorigenesis in several cancers, including breast cancer, non-small cell lung cancer, glioblastoma, renal carcinoma, and gall bladder cancer (Lao-On et al., 2018). Glycerol kinase is a highly conserved enzyme that functions at the junction of lipid synthesis and carbohydrate metabolism. This transferase catalyzes the transfer of a phosphorus group from ATP to glycerol and is a critical enzyme at the junction of fat and carbohydrate metabolism. Previous studies have shown that *Plasmodium falciparum* glycerol kinase catalyzes the ATP-dependent phosphorylation of glycerol to glycerol-3-phosphate (Naidoo and Coetzer, 2013). *Trypanosome* glycerol kinase catalyzes not only the forward reaction (ATP-dependent glycerol phosphorylation) but also the reverse reaction. Glycerol kinase is important for trypanosome survival and might be a promising drug target (Balogun et al., 2017). Succinate dehydrogenase is a functional member of both the Krebs cycle and the aerobic respiratory chain. Previous studies have shown that *C. sinensis* succinate dehydrogenase has varying activities during treatments with pyquiton, bithionol, and menichlopholan, and these activities are associated with morphological alterations of the tegument of the flukes (Hamajima et al., 1979; Pang et al., 1990). The determination of whether these bile duct fluke-specific enzymes play a key role in *C. sinensis* or *O. viverrini* requires further study. Some of the identified surface proteins of *C. sinensis*, such as Ras-related protein, beta-2-syntrophin, fatty-acid amide hydrolase, solute carrier family 25 and sodium/glucose cotransporter 4, were not found in other flukes and seem to be specific to *C. sinensis*. The functions of these outer surface proteins in *C. sinensis* have not been reported and are worth further research.

In this study, the proteins that might contribute to carcinogenesis were analyzed. Granulin, which is reportedly associated with carcinogenesis caused by *O. viverrini* (Smout et al., 2009), was identified in the *C. sinensis* tegument. In mammals, granulin is able to modulate cell growth (Shoyab et al., 1990; Culouscou et al., 1993; Zhou et al., 1993), and granulin is also a helminth-derived growth factor that causes the proliferation of mammalian cells (Smout et al., 2009, 2011). In addition, granulin was previously identified in the ESP supernatant fraction of *O. viverrini* after 24 h of culture (Mulvenna et al., 2010b). In *C. sinensis*, granulin was previously identified among the ESPs and is localized to the tegument and testes of the adult worm (Wang et al., 2017). In the current study, the absence of granulin in the supernatant obtained after 6 h of culture indicated that the protein is found at a low abundance among the ESPs. In addition to granulin, thioredoxin peroxiredoxin, carbonyl reductase 1 (CBR1) and cystatin might contribute to the carcinogenic process. Thioredoxin is a growth factor (Nguyen et al., 2006) that is overexpressed in many aggressive forms of cancer (Yoo et al., 2006). In *O. viverrini*, thioredoxin inhibits oxidative stress-induced apoptosis of bile duct epithelial cells, which indicates that liver fluke oxidoreductase might induce cholangiocarcinogenesis (Matchimakul et al., 2015). In *C. sinensis*, thioredoxin is localized to the tegument, vitelline gland, intestine, and intrauterine eggs of adult worms and is involved in the immunoregulation of the host immune response (Zhou et al., 2013), but the function of thioredoxin related to carcinogenesis remains unknown. Oxidoreductase-peroxiredoxin and carbonyl reductase 1 (CBR1) have been identified among the ESPs of *C. sinensis*, and both are overexpressed in liver cancer. The inhibition of CBR1 activity can enhance the effectiveness and decrease the cardiotoxicity of the anticancer drug anthracycline daunorubicin (DNR) in hepatocellular carcinoma (Huang et al., 2010). Cystatin is a superfamily of cysteine protease inhibitors that participates in various physiological and pathological processes. Cystatin stimulates interferon gamma-dependent nitric oxide production by macrophages (Vray et al., 2002), and two cystatins (Stifin-1 and Stifin-2) have been identified in *C. sinensis* (Kang et al., 2011, 2014). Cystatin might contribute to carcinogenesis at sites of inflammation via DNA damage and subsequent malignant transformation. Other proteins, such as FKBP12-rapamycin complex-associated protein, the molecular chaperone HtpG, phosphoenolpyruvate carboxykinase and growth factor receptor-binding protein 2, are predicted to play roles in the cancer pathway, but whether these proteins contribute to liver disease and liver cancer in *C. sinensis* infection needs further research. Liver fluke-induced CCA is a multifactorial pathological process associated with long-term infection and live fluke material-induced inflammation, and the release of carcinogenic substances by parasites is an important factor. The identified proteins will help elucidate the potentially important molecules and mechanisms of liver fluke-induced CCA.

## Conclusion

In the current study, we identified the ESPs and tegument proteome of *C. sinensis*, and these data could contribute to a more in-depth understanding of the complex parasite-host relationship, improve the diagnosis of clonorchiasis and benefit research on the pathogenesis and development of novel interventions, drugs and vaccines to control *C. sinensis* infection.

## Data Availability Statement

The datasets presented in this study can be found in online repositories. The names of the repository/repositories and accession number(s) can be found in the article/Supplementary Material.

## Ethics Statement

The animal study was reviewed and approved by The Institutional Review Board (IRB) approval by Guangxi Institutional Review Board (GXIRB) (IRB00001594).

## Author Contributions

YS, WZ, and ZJ contributed to the study conception and design. YS, KY, AL, YY, YH, WZ, ZJ, FO, and HW contributed to the sample collection and the acquisition, analysis and interpretation of the data. YS and KY contributed to the writing of the manuscript. All authors contributed to the article and approved the submitted version.

## Conflict of Interest

The authors declare that the research was conducted in the absence of any commercial or financial relationships that could be construed as a potential conflict of interest.

## Footnotes

1 http://www.ebi.ac.uk/GOA/

2 https://www.kegg.jp/

3 https://www.genome.jp/tools/kaas/

## Supplementary Material

Supplementary Table 1 The information of the identified excretory-secretory proteins.

Supplementary Table 2 The information of the identified tegument proteins.
